# Supplementary material for: The Characterization of a Novel PrMADS11 Transcription Factor from Pinus radiata Induced Early in Bent Pine Stem
Source: Int J Mol Sci. 2024 Jun 30;25(13):7245. doi: 10.3390/ijms25137245 (PMC11241540; doi:10.3390/ijms25137245)
Supplement: Supplementary file 1 [file ijms-25-07245-s001.zip › supplementary figures.pdf]

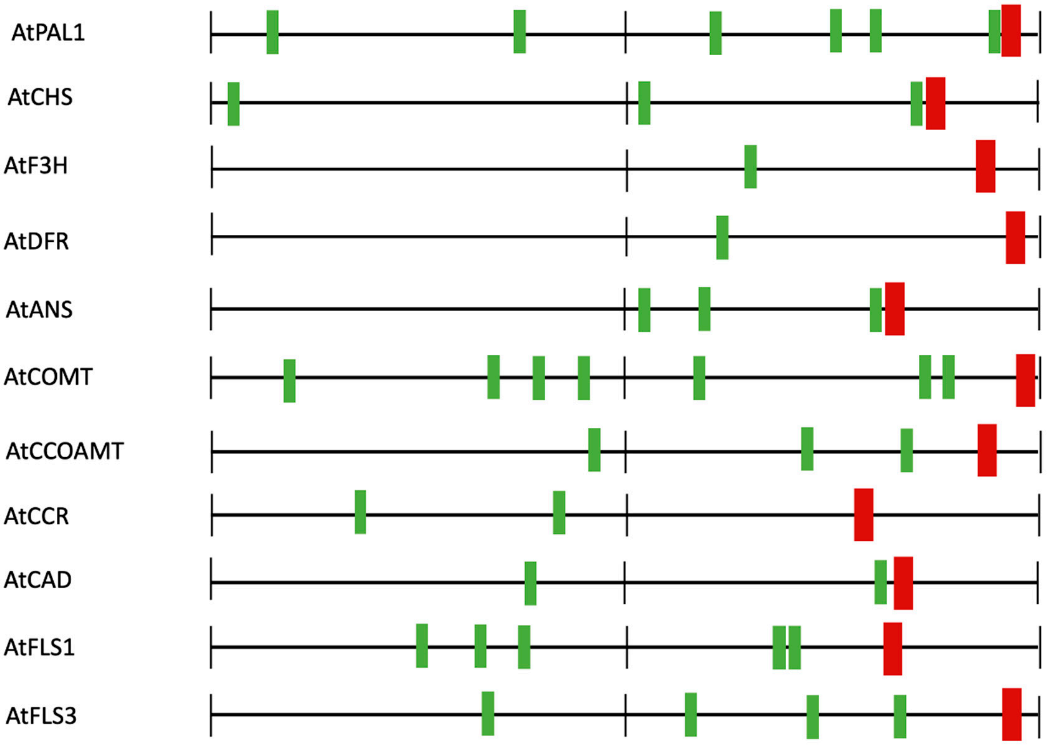

Figure S1. **Analysis of promotor region for several genes from the phenylpropanoid, monolignol and flavonoid synthesis pathway**. CARG *cis* elements for MADS TFs are shown in green. Different type of MADS from the MIRK type proteins can be linked to this DNA region like SEP-FLS-or SVP. TATA sequences are shown in red.
