# Supplementary material for: The Characterization of a Novel PrMADS11 Transcription Factor from Pinus radiata Induced Early in Bent Pine Stem
Source: Int J Mol Sci. 2024 Jun 30;25(13):7245. doi: 10.3390/ijms25137245 (PMC11241540; doi:10.3390/ijms25137245)
Supplement: Supplementary file 1 [file ijms-25-07245-s001.zip › Supplementary Tables.pdf]

Supplementary Table S1. List of protein sequences employed in the phylogenetic analyses of PrMADS11, including GenBank accession numbers.

MADS-box type	Id	Species	Gene bank accession
	AmSVP	*Amborella trichopoda*	XP_011629321
	AmSOC1	*Amborella trichopoda*	XP_01162901
	AmP3	*Amborella trichopoda*	BAD42444
	AtAGL1	*Arabidopsis thaliana*	NP_001190130
	AtAGL5	*Arabidopsis thaliana*	Q38847
	AtAGL6	*Arabidopsis thaliana*	NP_182089
	AtAGL24	*Arabidopsis thaliana*	NP_194185
	AtAGL28	*Arabidopsis thaliana*	NP_171660
	AtAGL33	*Arabidopsis thaliana*	AEC07824
	AtAGL38	*Arabidopsis thaliana*	AEE34356
	AtAGL57	*Arabidopsis thaliana*	AEE74037
	AtAGL75	*Arabidopsis thaliana*	AED94653
	AtAGL94	*Arabidopsis thaliana*	AEE34947
	AtSVP	*Arabidopsis thaliana*	AFU85632
	AtSEP	*Arabidopsis thaliana*	NP_564214
	BpSEP4	*Betula platyphylla*	QGP72146
	CaTM3	*Coffea arabica*	ADU56825
	CjAGL6	*Cryptomeria japonica*	BAG48496
	CjTM8-like	*Cryptomeria japonica*	BAG48494
	CjTM3-like	*Cryptomeria japonica*	BAG48497
	CjMADS1	*Cryptomeria japonica*	AAL05440
	CjMADS2	*Cryptomeria japonica*	AKE50811
	GbMADS1	*Ginkgo biloba*	AIC79629
	GbMADS2	*Ginkgo biloba*	AKE5081
	GbMADS3	*Ginkgo biloba*	BAD93167
	GbMADS4	*Ginkgo biloba*	BAD93168
	GbMADS5	*Ginkgo biloba*	BAD93169
	GbMADS6	*Ginkgo biloba*	QFP12054
	GbMADS7	*Ginkgo biloba*	BAD93171
	GbMADS8	*Ginkgo biloba*	BAD93172
	GbMADS9	*Ginkgo biloba*	AKE50810
	GbMADS10	*Ginkgo biloba*	BAD93174
	GbM5	*Ginkgo biloba*	AAM76208
	GpMADS3	*Gnetum parvifolium*	BAA85630
	GgM9	*Gnetum gnemon*	CAB44455

GgM11	*Gnetum gnemon*	CAB44457
MdAGL11	*Malus domestica*	NP_0012809318
OsMADS3	*Oryza sativa*	ACY26070
OsMADS47	*Oryza sativa*	AAQ23143
PkMADS1	*Paulownia kawakamii*	AAF22455
PaDAL10	*Picea abies*	AAQ13443
PaSOC1	*Picea abies*	AJA34534
PgMADS	*Picea gauca*	ATI36570
PrMADS1	*Pinus radiata*	AAD09206
PrMADS2	*Pinus radiata*	AAD09207
PrMADS3	*Pinus radiata*	AAB58907
PrMADS4	*Pinus radiata*	AAB80807
PrMADS5	*Pinus radiata*	AAB80808
PrMADS6	*Pinus radiata*	AAB80809
PrMADS7	*Pinus radiata*	AAB80810
PrMADS8	*Pinus radiata*	AAC27353
PrMADS9	*Pinus radiata*	AAC80806
PrMADS10	*Pinus radiata*	AKC96434
PrMADS11	*Pinus radiata*	AKC96435
PtDAL3	*Pinus tabuliformis*	AJP06319
PtDAL4	*Pinus tabuliformis*	AJP06277
PtDAL5	*Pinus tabuliformis*	AJP06278
PtDAL6	*Pinus tabuliformis*	AJP06279
PtDAL9	*Pinus tabuliformis*	AJP06280
PtMADS1	*Pinus tabuliformis*	AJP06319
PtMADS4	*Pinus tabuliformis*	AJP06277
PtMADS5	*Pinus tabuliformis*	AJP06323
PtSOC1	*Pinus tabuliformis*	AZA14799
PreMADS2	*Pinus resinosa*	AAD01266
PdelMADS1	*Populus deltoides*	ABV23568
PTM5	*Populus tormentosa*	ABG34340
PTSVP	*Populus tormentosa*	AGW52143
StMADS11	*Solanum tuberosum*	NP_001274754
StMADS16	*Solanum tuberosum*	NP_001275284
TbAGAMOUS	*Taxus bacata*	AEL13789

Supplementary Table S2. Probes used in EMSA assays.

Probe name	Sequence
AG	CTTATCCTTATAGAGAACAC
CARG1	CTTATCCATATTAGGACACC
CARG3(466)	AAATACTATATTTAGATCAA
CARG6(1555)	AAATCCATTATTATGAAAAC
MYB	

In red color the CArG-box of each sequence is shown.
